# Demonstrating transportability of real-world evidence: what do HTA bodies and health technology developers need?

**DOI:** 10.1017/S0266462326103924

**Published:** 2026-07-31

**Authors:** Ashley A. Jaksa, Blythe Adamson, Robert Szulkin, Christian Dehlendorff, Emma Hernlund, Vandana Ayyar Gupta, Melinda Hanisch, Jing Wang-Silvanto, Karen M. Facey

**Affiliations:** 1 Pedestal Health, USA; 2 University of Washington, USA; 3 Dental and Pharmaceutical Benefits Agency, Sweden; 4 Danish Medicines Council, Denmark; 5 National Institute for Health and Care Excellence, UK; 6 Merck & Co Inc, USA; 7 Astellas Pharmaceutical A/S, Finland; 8 RWE4Decisions Secretariat, FIPRA, Belgium; 9 University of Edinburgh, United Kingdom

**Keywords:** real-world evidence, transportability, health technology assessment, decision-making, external validity, comparative effectiveness research

## Abstract

Real-world evidence (RWE) is reshaping how health technology assessment (HTA) bodies evaluate therapies, yet a critical gap persists: while transportability methods enable researchers to adapt treatment effect estimates from RWE studies in another jurisdiction to local contexts, the field remains unclear on when these analyses impact decision-making. This article synthesizes insights from HTA bodies, health technology developers (HTDs), and methodologists to clarify what is missing from today’s transportability conversation. We find that methodological progress has outpaced guidance on its utility; HTDs risk investing in transportability analyses without clear evidence that they reduce key uncertainties or affect recommendations. We identify three fundamental gaps: the absence of objective thresholds for determining whether transportability assumptions are adequately met; limited guidance on when quantitative transportability is needed versus qualitative assessment; and variation in HTA bodies’ evaluation and application of transported evidence. These gaps create a critical impasse where HTDs must decide whether to invest in such analyses, while HTA bodies lack standardized frameworks for their assessment. We argue that transportability cannot transition from academic exercise to a decision-making tool without explicit early dialogue between stakeholders, transparent reporting standards, and capacity building within HTA bodies. We present actionable recommendations to build HTA expertise, create a case study repository, and define contexts where transported RWE demonstrably impacts patient access. As evidence dossiers increasingly rely on nonlocal data, addressing these strategic and operational gaps becomes essential to help patients.

## Introduction

The push for appropriately governed secondary data use is accelerating as healthcare stakeholders seek to harness diverse health system datasets to optimize patient care (1). Health technology developers (HTDs) and health technology assessment (HTA) bodies are utilizing real-world evidence (RWE) to inform clinical assessments and economic models (2–6). For example, descriptive RWE studies are used to characterize the local population, care and treatment pathways, qualitatively evaluate the external validity of clinical trial findings for the HTA body’s population of interest, and verify assumptions in economic modeling (7;8). In rarer instances, real-world data (RWD) is used as an external control to estimate comparative efficacy or benchmark trial results (9;10). RWE can also be used post-launch to evaluate a range of decision-relevant uncertainties (11–13).

A major challenge in the use of RWE is internal validity (i.e., the extent to which the results of an RWE study accurately reflect the true situation in the specific population being studied, free from systematic errors (bias) or the effects of chance (14)). Since RWE comparative effectiveness studies are not randomized, researchers must control for potential confounding to demonstrate the true effect of a therapy. Substantial work has outlined best practices for addressing internal validity challenges (e.g., HTA body guidance). External validity (or “the extent to which the results of a given study can be applied outside the original study context, such as to another target population of interest” (15)) is another key challenge, but has been given less attention.

Although local evidence is ideal, HTA bodies are increasingly receiving submissions that include RWE generated from other countries (16). This is often driven by the lack of high-quality local data for the specific clinical application or due to small sample sizes for rare conditions and/or small populations or limited HTA capacity. RWE is recognized for its superior external validity, as it incorporates diverse patient populations and reflects contemporary clinical practice. However, when such studies are conducted outside the target jurisdiction, HTA bodies may question the relevance of the findings for their specific local population.

**Figure 1.**
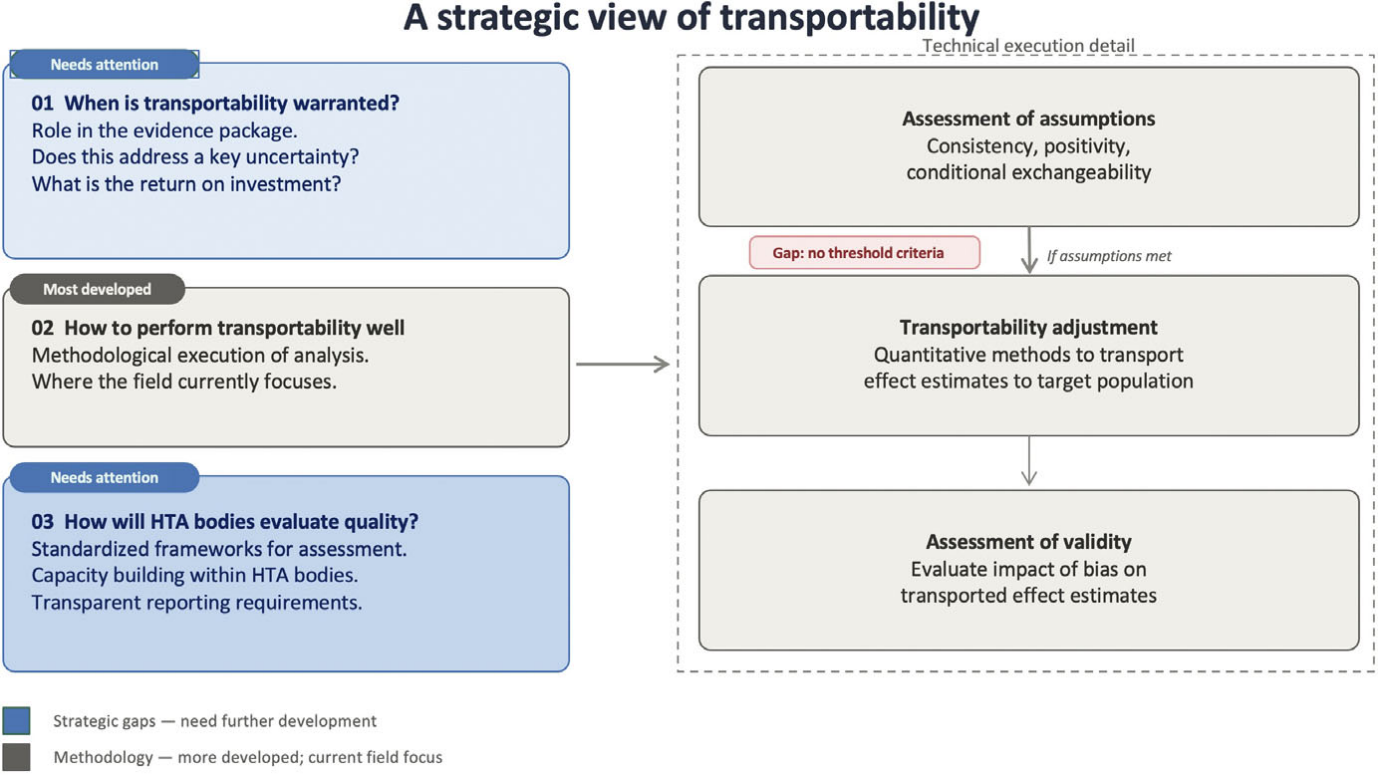
Pivoting the focus from transportability methods to their strategic use. Figure 1. long description.

Advances in RWE methodology provide sophisticated methods to address external validity concerns and assist HTDs and HTA bodies in “transporting” effect estimates to local settings. **Transportability** refers to how well inferences generated from a sampled population may extend to a target population from which the original sample was not derived (17). Transporting effect estimates can be done with multiple study types, like randomized controlled trials (RCTs) or RWE studies. In 2026, an International Society for Pharmacoepidemiology (ISPE) working group operationalized transportability analysis into a three-step process, which includes (1) evaluating the feasibility and appropriateness of the transportability analysis (i.e., assuring the assumptions are met), (2) selecting the optimal statistical method to transport the effect estimates, and (3) applying analytical approaches (e.g., sensitivity analysis and qualitative bias analysis) to ensure the trustworthiness of the transported treatment effects ((18); Figure 1). Throughout this article, “transportability analysis” is defined as the comprehensive three-stage process; where necessary, individual components are referred to specifically as the assessment of assumptions, quantitative adjustment, or validation of results.

**Figure 2.**
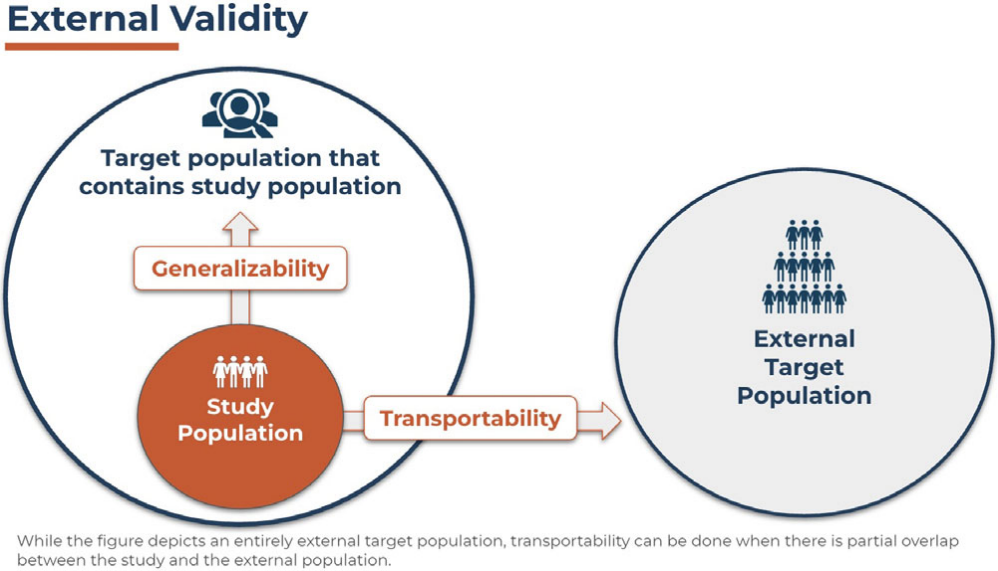
Defining the relationship between generalizability and transportability. While the figure depicts an entirely external target population, transportability can be done when there is partial overlap between the study and the external population. Figure 2. long description.

Transportability is a form of external validity, which is the ability of a study design “to provide findings that can be generalized to other populations, contexts, and periods” (19), such as another clinical situation or geographical region. While often used interchangeably with generalizability, we distinguish between these two concepts for this article. Generalizability refers to the extension of findings from a study sample to a broader target population from which that sample was originally drawn (i.e., the study population is a representative subset). In contrast, transportability concerns the application of findings to a distinct, external population that was not part of the original sampling frame (Figure 2) (18;20).

When a high-quality RWE study from a different jurisdiction addresses a gap in the clinical research, transportability analyses have the potential to boost an evidence submission by improving the evidence’s relevance to the decision problem (15). While several HTA bodies have stated an openness to evaluating nonlocal data within their RWE guidance (21) and methodological best practices for transportability analysis have been recently outlined (18), it is still unclear when HTA bodies might accept transportability analyses, in terms of types of assessment, analytical approaches, and reporting requirements.

This article aims to outline the current state of transportability research, identify key strategic and operational gaps in its implementation for HTA, and provide actionable recommendations for HTDs and HTAs to promote its wider, appropriate implementation. Work to date has primarily focused on transportability methodology and applying these methods to real-world examples through demonstration projects. Recently, Gupta et al. (16) provided a valuable conceptual primer on transportability and its relevance to HTA; however, this current article moves beyond the methodological foundations to synthesize multi-stakeholder perspectives and identify the strategic and operational gaps that must be addressed before transportability can meaningfully influence HTA decision-making.

### Sources

The insights presented in this article are drawn from two primary sources. First, an RWE4Decisions (22) learning network roundtable held in May 2025, which convened 52 participants from HTA bodies in Belgium, Bulgaria, Canada, Denmark, England, Germany, the Netherlands, and Sweden, HTDs, academics/analytic experts, and patient experts to discuss whether, how, and when transportability analyses should be undertaken to inform HTA. This included presentation of three transportability case studies from different stakeholders, which led to the development of the hypothetical example shown in this article. Second, a review of recent literature on transportability methodology, HTA evidence standards, and RWE assessment frameworks informed this article. No formal systematic search was conducted; literature was identified through expert knowledge and citation tracking, which may have introduced some selection toward more prominent or recently published work. The recommendations were developed iteratively by the authors.

### Current state – a focus on transportability analysis methods

Most work on transportability has focused on methodology, outlining assumptions necessary for transportability and illustrating how to apply these methods through demonstration projects. Multiple literature reviews (15;23) have cataloged different transportability adjustment methods (e.g., weighting, outcome regression, and combined approaches) and outlined the specific assumptions necessary for their application (15;16;21;24). First, the foundational assumption is that the study that will be transported has internal validity (e.g., an RWE study of high quality and built using fit-for-purpose data). Once that has been established, the assumptions necessary for transportability can be explored. As transportability analyses involve an external target population, ensuring consistency, positivity, and conditional exchangeability is essential, as outlined in Table 1.

**Table 1.** Key requisites for transportability Table 1. long description.

**Consistency**	Patients in both the study and target population receive the same version of the treatment/intervention (also known as the stable unit treatment value assumption) (e.g., treatment pathways in both countries are similar)
**Positivity**	The patient characteristics (e.g., covariate patterns) in the study are similar to those in the external target population
**Conditional exchangeability**	Any relevant differences between populations can be measured and adjusted for

*Note:* Definitions in this table are intentionally simplified for accessibility. Readers seeking formal technical definitions are encouraged to consult the ISPE best practices guidance (Reference 18) for precise statistical formulations of each assumption.

Historically, transportability case studies have primarily focused on transporting RCT treatment effects to external real-world populations (15;23), though recently there has been an increase in demonstration projects transporting RWE treatment effect estimates to external real-world populations (6;25;26). These case studies have increased our understanding of how transportability analysis can be used for RWE studies, and the work necessary to justify that the assumptions have been met. The foundational work documenting methods and assumptions, and applying these methods in demonstration projects, has led ISPE to develop transportability analysis best practices (18). Even with this best practice guidance, methodological gaps still exist.

### Methodological gaps

The transportability assumptions (i.e., consistency, positivity, and conditional exchangeability, Table 1) are rarely black and white; they exist in a gray area where a strategic go/no-go decision must be made. For instance, the conditional exchangeability assumption requires that all relevant differences between the RWE study population and the target population are measurable in the data and thus can be adjusted for (e.g., effect modifiers). However, in RWE studies – which are built from data not collected for research purposes – it is nearly impossible to capture all potential confounders. Esnault et al. (6) demonstrated this challenge when transporting overall survival estimates from the United States to France for patients with metastatic triple-negative breast cancer. The authors noted that some prognostic factors, such as certain socioeconomic factors, were not fully captured in the RWD in France and could not be adjusted for. While this was acknowledged as a limitation, the authors postulated that these unmeasured factors would probably have a limited impact on the transported results and therefore considered the conditional exchangeability assumption met. If the unmeasured confounders were more prominently associated with survival (e.g., stage and profile of disease, performance status, and prior treatments), the decision to proceed with transportability analysis may have been different. Currently, there are no documented thresholds for definitively determining whether the assumptions are met, and researchers must make judgments to proceed with transportability. Further exploration of assumption thresholds is needed, and lessons learned from several demonstration projects are necessary to articulate clearer criteria for meeting these critical assumptions and signaling that transportability analysis is “a go.”

### Gaps in strategic considerations for implementing transportability in HTA

Beyond the technical and methodological considerations of *whether* a transportability analysis is appropriate and *how* to perform robust transportability analyses, there remains a challenge about *when* it should be done and how the analysis will influence HTA decision-making. Transportability analysis is not a “Swiss Army Knife”; its application is not universally useful across all situations. This section explores our understanding of the larger strategic context, when to apply these methods in the context of HTA, followed by an examination of how a transportability analysis will be evaluated for quality and relevance by HTA bodies (Figure 1).

#### When is transportability analysis warranted?

Cases where the transportability of RWE treatment effect estimates is relevant often follow the use cases for RWE in general. For example,

- In rare diseases, when RCTs are not ethical or feasible. In these instances, RWD is often used as an external control arm to provide effect estimates compared to the single-arm study. If the external control is not built using local data, transportability analysis could be beneficial for estimating the treatment effect in the population of interest.
- Other use cases relevant to HTA decision-making include instances where the RCT evidence does not utilize the most appropriate SoC or in crowded markets where the RCT evidence lacks head-to-head comparisons.

In both these instances, RWE studies evaluating treatment effects can be beneficial. However, if the RWE studies were not built using local data, transportability analysis can provide important context on the treatment effects in the population of interest.

While these use cases are areas where transportability adjustment may be appropriate, conducting a full transportability analysis (especially the qualitative assessment of assumptions and the adjustment) requires resources, sufficient data to address key confounders, and time. Thus, HTDs must decide when to invest in transportability analysis, ideally in collaboration with HTA bodies addressing three core questions:

- What role will the transported RWE treatment effect estimates play in the evidence submission?
- Could the transported RWE treatment effect estimates address a key uncertainty identified by the HTA body?
- Is it likely that the transported RWE treatment effect results ultimately impact the final decision-making process?

Furthermore, HTDs must address internal strategic and logistical questions:

- Can a simpler qualitative external validity assessment stand alone and provide confidence that the RWE results can apply to the external population?
- If not, is the potential impact of the transportability analysis worth the time and effort to undertake the work?
- Is the transportability analysis feasible within the HTA submission timeline? Which markets should be prioritized, and are certain country pairs more likely to yield acceptable results (e.g., Sweden to Denmark vs. Sweden to Spain).

Consider the hypothetical example provided in **Box 1**.

Box 1. Hypothetical use of transportability in HTA decision-making.

An evidence submission for therapy Z was submitted for initial reimbursement approval to Sweden’s HTA body, TLV. The evidence submission included a phase III RCT that demonstrated statistically significant and clinically meaningful improvement for the intervention group receiving therapy Z compared to the placebo group. However, placebo/no treatment was not the relevant comparator specified by TLV. A major uncertainty was how therapy Z compares to the Swedish SoC. The HTD considered an indirect treatment comparison of RCTs for therapy Z and the SoC, but the heterogeneity across the trials made the indirect treatment comparison infeasible. Since therapy Z was already utilized in the US, the HTD proactively included in the initial submission a US-based RWE study that compared therapy Z to the US SoC – which happened to be similar to the SoC used in Sweden. Local RWD is unavailable to support a Swedish RWE study, and due to key differences between the US and Swedish patient populations and healthcare systems, TLV questioned the external validity of the US RWE findings. To address this gap, the HTD performed and resubmitted a transportability analysis, transporting the effect estimates from the US-based RWE study to the Swedish population. The results provided Swedish-population estimates of therapy Z’s effect versus SoC for use as effectiveness evidence and cost-effectiveness model inputs.

In the hypothetical example, TLV is likely to qualitatively assess the generalizability of the RCT and RWE results. For example, assessing whether the patients included in the RCT and US-based RWE study are representative of the Swedish population. In some cases, TLV uses Swedish RWD data to aid that assessment, though it remains high-level and qualitative. If the HTD provided a transportability analysis that transported the effect estimates, it could be valuable. However, because many HTA assessors are not familiar with this transportability analysis, questions will be raised, and acceptance of such methods may be more challenging.

Let us consider the questions above in the context of the hypothetical example in Box 1 and, for simplicity, assume that the US RWE study was high quality, the key assumptions of transportability were met, and the results were robust. Considering the totality of evidence, the US-based RWE study and the transported effect estimate likely play a supporting role to the placebo-controlled RCT. The US-based RWE study provides important context for therapy Z’s performance compared to the Swedish SoC, which is not covered by the comparison with placebo in the RCT; the transported RWE provides additional effectiveness context specifically for the Swedish population. This raises the central question: Are the transported RWE effect estimates a *nice-to-have* or are they truly *necessary* for TLV’s decision-making?

Specifically, the HTD must assess:

- If the transported effect estimates were omitted, would this result in an adverse access outcome (e.g., a “do not recommend” decision), and
- Whether the potential benefit of the transportability analysis outweighs the finite costs and resources dedicated to completing the transportability analysis for the TLV submission.

Finally, given that TLV is only one jurisdiction, the HTD must strategically determine how to prioritize similar transportability analyses across other target markets or discuss when an analysis could be used in another similar HTA jurisdiction. With limited case studies of transported RWE effect estimates in HTA decision-making, there is no data-driven way to address these questions.

#### How will HTA bodies evaluate the quality of transportability analysis?

Due to the limited track record of transportability assessments in HTA decision-making and limited guidance from HTA bodies and regulators on when and how to complete transportability analysis, it remains unclear how HTA bodies will evaluate the quality of these complex analyses. The HTA body’s initial hurdle is evaluating the internal validity of the original RWE study – a challenge in itself. Research has consistently shown variability in how HTA bodies assess RWE study quality, often accompanied by limited public documentation or the factors leading to evidence acceptance (27;28). Furthermore, successfully evaluating transportability analysis requires specialized methodological expertise within the HTA body. Ultimately, the lack of standardized RWE assessment, combined with the technical demands of evaluating transportability analysis, presents a significant barrier to consistent implementation.

### Recommendations

While methods for transporting effect estimates exist and best practices outline how to “do it well,” there is still considerable uncertainty regarding transportability’s impact on HTA decision-making (i.e., when it is appropriate and when it will inform decision-making) and how HTA bodies will evaluate the quality of the analysis. Understanding the contextual and strategic considerations of transportability requires further development. Our recommendations for a path forward include:

#### Back to basics

Transportability analysis begins with a qualitative evaluation of consistency, positivity, and conditional exchangeability, which is similar to an HTA body’s qualitative assessment of the external validity of the submitted evidence. While transportability methods continue to mature and HTA bodies’ comfort levels with RWE evaluation rise, HTDs may be able to progress by focusing on the initial steps of transportability. A foundation of an HTA body’s critical assessment of clinical effectiveness has always been to consider the external validity of the submitted evidence. This requires qualitatively assessing how treatment patterns, care pathways, prevalence/incidence, patient demographics, and clinical characteristics compare between the study and the local context. These core elements are, fundamentally, the underlying assumptions of transportability. When questions of external validity arise, HTDs and HTA bodies should first focus on qualitative analysis of the comparability of patient populations. If there are no reasons to expect the outcomes to be different, then an unadjusted analysis may be preferred, and transportability adjustment is not necessary. Anchoring and agreeing on these foundational components will effectively illustrate when complex transportability methods are actually warranted, potentially saving significant time and effort.

#### Build confidence by starting with transporting RCT effect estimates

Transportability analysis can be done with a wide variety of studies that evaluate treatment effects, and HTA bodies are familiar with and comfortable in evaluating RCTs. By prioritizing RCT-to-jurisdiction transportability analysis, HTA bodies can focus on the transportability methodology itself without concerns associated with RWE given their differing capabilities in this field.

#### Importance of scientific advice

HTDs considering a transportability analysis, or HTA bodies proposing it, must clearly demonstrate how transporting RWE effect estimates to local populations will improve decision-making. While additional evidence is often beneficial, investing in transportability analyses is unlikely to be undertaken unless the path to impact is explicitly clear. Early dialogue and close alignment between HTDs and HTA bodies on the impact of these transported results is key, as it directly informs the HTD’s internal risk/benefit analysis for completing the time-intensive and expensive work. HTDs can benefit from discussions with national HTA bodies on their individual evidence needs (29). However, there are challenges with scientific advice, as not all HTA bodies offer it, and those that do have limited capacity.

#### Transparency

HTA bodies require transparent reporting of RWE studies from local and nonlocal data sources, whether or not transportability analysis has been undertaken (4). The creation of the RWD study itself is prone to many choices that could introduce bias, and the application of complex transportability analysis could compound the problem. Transparency in reporting the underlying RWE study quality, the rationale for transportability, fulfillment of underlying assumptions, adjustment methods, validation, and results becomes essential. Transparency for transportability analysis should be no different than transparency recommendations for RWE causal interference studies (4). Similarly, HTA bodies need to be transparent with HTDs regarding how the transported evidence will be used in decision-making and how it is evaluated.

#### HTA body capacity building

HTA staff need to develop the technical capabilities required to assess RWE and advanced methodologies, such as transportability analysis. Globally, RWD infrastructure is undergoing structural evolution – particularly in Europe, where initiatives such as the European Health Data Space and federated networks like DARWIN EU are improving data interoperability and the availability of RWD needed to satisfy transportability assumptions. HTA bodies must proactively expand their expertise to evaluate the resulting influx of RWE submissions. HTA bodies should also consider providing more detailed guidance on when transported RWE results are relevant, how transportability will be assessed, and ultimately, how this evidence will be considered in decision-making. We recognize that “proscriptive” guidance (e.g., a recipe for when and what transportability methods are most appropriate in different use cases) is not attainable. HTA bodies should consider high-level guidance (e.g., adopting ISPE’s best practices), plus thorough analysis and commentary on case studies, which could provide more detail on what the HTA body considers a good use of transported estimates and how they will analyze its quality.

#### Additional demonstration projects and case studies

To determine where transportability analyses are likely to be most influential, it is instructive to critically review past cases. The most instructive lessons on methodology often come from case studies where the methods were unsuccessful (30); in the case of transportability, this could be where one or more methodological assumptions proved untenable. Examining successful and unsuccessful case studies is crucial for understanding both methodological challenges and contextual considerations. For instance, a failure due to irreconcilable differences in clinical practice (a violation of the consistency assumption) highlights the paramount importance of clinical alignment between countries. By systematically documenting and analyzing the reasons for success and failure – whether methodological or strategic – HTDs and HTA bodies can develop a more nuanced framework for the “go/no-go” decision, identifying early signs that a transportability analysis may be valuable. Also of value are collaborative demonstration projects with HTA bodies and HTDs, which can help illustrate when transportability analysis makes sense and supports decision making compared to doing transportability as an academic exercise to prove methods work.

## Discussion

Our review and insights from the RWE4Decisions roundtable confirm that substantial work has been completed in defining the methodology for transportability, including the underlying assumptions (consistency, positivity, and conditional exchangeability) and structured, stepwise best practices for analysis. However, the current state of implementation reveals a crucial gap: the community has mainly focused on the assumptions and methods relevant for transportability analysis, but not adequately on when and why it is warranted to support HTA decision-making. In short, the field lacks clarity on when transportability is likely to influence decision-making.

In the near term, the strategic focus must shift toward defining the optimal reimbursement contexts for transportability analysis. This includes clarifying how transported treatment effects integrate into the evidence submission, their utility in mitigating key evidentiary uncertainties, and their ultimate influence on HTA reimbursement decisions. We posit that methodological clarity will emerge through an increased volume of transportability analysis in submissions, demonstration projects, and dialogue between HTA bodies and HTDs. Currently, the absence of robust HTA guidance and a paucity of case studies create an ill-defined return on investment (ROI) for HTDs. Given that transportability analysis is both time- and resource-intensive, the lack of a predictable ROI discourages investment, thereby limiting HTA bodies’ exposure to these methods. A collaborative framework between HTA bodies and HTDs is essential to break this circular problem.

Several stakeholders play a role in generating better RWE for HTA decision-making (31). Analytics groups and academics have paved the way forward by developing and adapting transportability frameworks and methodologies. However, methodological innovation alone is not sufficient to ensure meaningful uptake. HTA bodies and HTDs each contribute essential expertise that determines whether transported evidence can be generated, evaluated, and ultimately used in decisions. HTA bodies shape evidentiary standards that signal when transportability is useful; HTDs determine whether the investment will produce actionable reductions in uncertainty. Together, they must move beyond methodological refinement toward shared expectations, transparent reporting, and collaborative case-based learning to ensure transportability evolves into a practical tool that improves patient access.

This study has limitations. While multi-stakeholder in composition, the roundtable may not have fully represented all relevant perspectives (e.g., lower- and middle-income countries). Second, the literature informing this study was identified through expert knowledge and citation tracking rather than a formal systematic search, which may introduce selection bias toward more prominent or recently published work. Third, the recommendations presented reflect the authors’ synthesis and interpretation rather than formal consensus and may not fully capture the range of views expressed during the roundtable.

## Conclusion

This article has outlined the current state of transportability research, identified strategic and operational gaps in its implementation for HTA, and proposed actionable recommendations for stakeholders to advance its appropriate use. While transportability methodologies are mature and their conceptual value is widely acknowledged, the field now requires pragmatic approaches, case-based evidence, and capacity building to ensure that transported RWE can meaningfully inform HTA decisions. As reliance on nonlocal RWD increases and as health systems continue to adopt more diverse data sources, the need for structured, transparent, and context-specific approaches to transportability will only grow. Advancing this work is essential not only for improving methodological rigor but also for ensuring that patients benefit from timely and equitable access to effective therapies supported by the best available evidence.

## Long descriptions

### Figure 1. Long description

The bottom box is headed, Assessment of validity, with sub-text: Evaluate impact of bias on transported effect estimates.

The middle box is headed, Transportability adjustment, with sub-text: Quantitative methods to transport effect estimates to target population.

The right technical workflow panel shows three gray boxes arranged vertically and connected by downward-pointing arrows. The top box is headed, Assessment of assumptions, with sub-text: Consistency, positivity, conditional exchangeability. The first box is connected by a downward arrow to the middle box, Transportability adjustment, with text right of the arrow noting “if assumptions are met”. Between the first and second boxes, a red pill-shaped label reading, Gap: no threshold criteria, branches to the left of the connecting arrow.

The second box is gray, labeled, Most developed, and is headed, 02 How to perform transportability well. It lists two items: Methodological execution of analysis; Where the field currently focuses. A horizontal arrow connects this box rightward to the technical workflow panel.

The third box is dark blue, labeled, Needs attention, and is headed, 03 How will HTA bodies evaluate quality? It lists three items: Standardized frameworks for assessment; Capacity building within HTA bodies; Transparent reporting requirements.

The first box is dark blue, labeled, Needs attention, and is headed, 01 When is transportability warranted? It lists three items: Role in the evidence package; Does this address a key uncertainty?; What is the return on investment?

The left panel contains three vertically stacked boxes, color-coded using two categories defined in a legend at the bottom of the figure: dark blue boxes indicate, Strategic gaps – need further development; gray boxes indicate, Methodology – more developed; current field focus.

The figure is titled, A strategic view of transportability. It is divided into a left panel with three strategic question boxes and a right panel containing a technical workflow within a dashed border labeled, Technical execution detail.

Navigate back to Figure 1..

### Figure 2. Long description

A footnote in gray text at the bottom of the figure reads: While the figure depicts an entirely external target population, transportability can be done when there is partial overlap between the study and the external population.

The right circle is large, filled with light gray, and outlined in dark navy blue. It contains a triangular arrangement of small person icons in dark navy blue and is labeled, External Target Population, in bold dark text.

A second orange-outlined box labeled, Transportability, sits to the right of the inner orange circle, between the two main circles. A rightward-pointing arrow leads from the inner circle toward the right circle, indicating the direction of inference.

An orange-outlined box labeled, Generalizability, sits between the inner orange circle and the upper portion of the outer navy circle. An upward-pointing arrow leads from the inner circle toward the, Target population that contains study population, label, indicating the direction of inference.

Within the lower portion of this large circle sits a smaller, filled circle in dark orange-red. It contains a smaller group icon and is labeled, Study Population, in bold white text.

The diagram contains two circles. The left circle is large, outlined in dark navy blue, and represents the, Target population that contains study population, as labeled in bold text at the top of the circle, accompanied by a group icon.

The figure is titled, External Validity, in bold dark text with a short orange underline.

Navigate back to Figure 2..

### Table 1. Long description

The table consists of two columns and three rows of data.

* Row 1: The first column is Consistency. The second column defines it as patients in both the study and target population receiving the same version of the treatment or intervention, also known as the stable unit treatment value assumption. An example provided is that treatment pathways in both countries are similar.

* Row 2: The first column is Positivity. The second column defines it as patient characteristics, such as covariate patterns, in the study being similar to those in the external target population.

* Row 3: The first column is Conditional exchangeability. The second column defines it as any relevant differences between populations being able to be measured and adjusted for.

Navigate back to Table 1..

## References

[1] European Health Data Space European Health Data Space. [Internet]. [cited 2026 Mar 14]. Available from: https://healthdataspace.eu

[2] Facey KM, Rannanheimo P, Batchelor L, Borchardt M, de Cock J. Real-world evidence to support payer/HTA decisions about highly innovative technologies in the EU-actions for stakeholders. Int J Technol Assess Health Care. 2020;3:1–10. 10.1017/S026646232000063X 32878663

[3] Capkun G, Corry S, Dowling O, et al. Can we use existing guidance to support the development of robust real-world evidence for health technology assessment/payer decision-making? Int J Technol Assess Health Care. 2022;38(1):e79. 10.1017/S0266462322000605 36321447

[4] CDA-AMC. Guidance for Reporting Real-World Evidence [Internet]. [cited 2026 Mar 5]. Available from: https://www.cda-amc.ca/guidance-reporting-real-world-evidence

[5] Jaksa A, Arena PJ, Hanisch M, Marsico M. Use of real-world evidence in health technology reassessments across 6 health technology assessment agencies. Value Health. 2025;28(6):898–906. 10.1016/j.jval.2025.02.012. 40054770

[6] Esnault C, Simomia L, Machuron G, Robain M, Adamson B, Machuron V. Transportability of overall survival estimates from the USA to France in patients with metastatic triple-negative breast cancer. ESMO Real World Data Digital Oncol. 2025;10:100648. 10.1016/j.esmorw.2025.100648

[7] Claire R, Elvidge J, Hanif S, et al. Advancing the use of real world evidence in health technology assessment: insights from a multi-stakeholder workshop. Front Pharmacol. 2024;14:14. 10.3389/fphar.2023.1289365 PMC10811058 38283835

[8] Murphy LA, Akehurst R, Cunningham D, de PG, Solà-Morales O. Real-world evidence to support health technology assessment and payer decision making: is it now or never? Int J Technol Assess Health Care. 2025;41(1):e20. 10.1017/S0266462325000145 40162485 PMC12018852

[9] Vidalis A, Dumoulin O, Godbole M, Proenca CC. The role and value of real-world evidence in health technology decision-making in France, Germany, Italy, Spain, and the UK: insights on external control arms. Int J Technol Assess Health Care. 2025;41(1):e25. 10.1017/S0266462324004720 40260460 PMC12019763

[10] Schuster V. Real-world evidence requirements with respect to external control arms of single-arm clinical trials by major regulatory and HTA agencies – comparative review of guidelines and identification of trends based on seven recent case studies. BJSTR. 2024;58(1). 10.26717/BJSTR.2024.58.009096

[11] Facey KM, Espin J, Kent E, et al. Implementing outcomes-based managed entry agreements for rare disease treatments: nusinersen and tisagenlecleucel. PharmacoEconomics. 2021;39(9):1021–1044. 10.1007/s40273-021-01050-5 34231135 PMC8260322

[12] Kent S, Meyer F, Pavel A, et al. Planning post-launch evidence generation: lessons from France, England and Spain. Clin Pharmacol Ther. 2025;117(4):961–966. 10.1002/cpt.3586 39960266 PMC11924153

[13] Greco A, Frederix GWJ, Hooft L, Ten Ham RMT. A systematic review of challenges and opportunities in the implementation of managed entry agreements for advanced therapy medicinal products. Clin Ther. 2025;47(2):e16–e26. 10.1016/j.clinthera.2024.11.019 39706763

[14] Caparrotta TM, Dear JW, Colhoun HM, Webb DJ. Pharmacoepidemiology: using randomised control trials and observational studies in clinical decision-making. Br J Clin Pharmacol. 2019;85(9):1907–1924. 10.1111/bcp.14024 31206748 PMC6710512

[15] Levy NS, Arena PJ, Jemielita T, et al. Use of transportability methods for real-world evidence generation: a review of current applications. J Comp Eff Res. 2024;13(11):e240064. 10.57264/cer-2024-0064 39364567 PMC11542082

[16] Gupta A, Duffield S, Shephard C, Ting E, Popat S, Cheung WY Transportability of nonlocal real-world evidence and its relevance to health technology assessment: a primer. J Comp Eff Res 2025 Oct;14(10):e250041. doi:10.57264/cer-2025-0041 40836909 PMC12465918

[17] SUSTAIN-HTA SUSTAIN-HTA Taxonomy tool [Internet]. [cited 2026 Mar 14]. Available from: https://sustain-hta.shinyapps.io/SUSTAIN-Taxonomy/

[18] Adamson B, Remior-Azocar A, Lui W, et al. Transportability analyses in comparative effectiveness research: a conceptual framework and methodological principles endorsed by the International Society for Pharmacoepidemiology. Pharmacoepidemiol Drug Saf. 2026;35(6):e70396. 10.1002/pds.70396 42144278 PMC13180502

[19] HtaGlossary.net HTA Glossary [Internet]. [cited 2026 Mar 14]. Available from: http://htaglossary.net/HomePage

[20] Dahabreh IJ, Hernán MA. Extending inferences from a randomized trial to a target population. Eur J Epidemiol. 2019;34(8):719–722. 10.1007/s10654-019-00533-2 31218483

[21] Jaksa A, Arena PJ, Chan KKW, Ben-Joseph RH, Jónsson P, Campbell UB. Transferability of real-world data across borders for regulatory and health technology assessment decision-making. Front Med (Lausanne). 2022;9:1073678. 10.3389/fmed.2022.1073678 36465931 PMC9709526

[22] RWE4Decisions RWE4Decisions. [Internet]. [cited 2026 Mar 14]. Available from: https://rwe4decisions.com/

[23] Turner AJ, Sammon C, Latimer N, et al. Transporting comparative effectiveness evidence between countries: considerations for health technology assessments. PharmacoEconomics. 2024;42(2):165–176. 10.1007/s40273-023-01323-1 37891433 PMC10811184

[24] Clunie-O’Connor C, Thuresson PO, Masters E, et al. Crossing borders: the need for empirical evidence of real-world evidence transportability in oncology. J Comp Eff Res. 2025;14(8):e250053. 10.57264/cer-2025-0053 40637151 PMC12308535

[25] Vuong Q, Metcalfe RK, Ling A, Ackerman B, Inoue K, Park JJ. Systematic review of applied transportability and generalizability analyses: a landscape analysis. Ann Epidemiol. 2025;104:61–70. 10.1016/j.annepidem.2025.03.001 40064249

[26] Kent S, Mpofu P, Duffield S, et al. Evaluating transportability of overall survival estimates from US to UK populations receiving first-line treatment for advanced non-small cell lung cancer: a retrospective cohort study. BMJ Open. 2024;14(12):e085722. 10.1136/bmjopen-2024-085722 PMC11628978 39645255

[27] Jaksa A, Louder A, Maksymiuk C, et al. A comparison of 7 oncology external control arm case studies: critiques from regulatory and health technology assessment agencies. Value Health. 2022;25(12):1967–1976. 10.1016/j.jval.2022.05.016 35760714

[28] Candore G, Martin C, Mills MJ, et al. FRAME: framework for real-world evidence assessment to mitigate evidence uncertainties for efficacy/effectiveness – an evaluation of regulatory and health technology assessment decision making. Clin Pharmacol Ther. 2025;118(3):649–661. 10.1002/cpt.3713 40493357 PMC12355015

[29] Annemans L, Makady A. TRUST4RD: tool for reducing uncertainties in the evidence generation for specialised treatments for rare diseases. Orphanet J Rare Dis. 2020;15:127. 10.1186/s13023-020-01370-3 32456653 PMC7251888

[30] Wang SV, Schneeweiss S, RCT-DUPLICATE Initiative, et al. Emulation of randomized clinical trials with nonrandomized database analyses. JAMA. 2023;329(16):1376–1385. 10.1001/jama.2023.4221 37097356 PMC10130954

[31] Jaksa AA, Pavel AN, Aapro M, et al. Actions for stakeholders to develop better real-world evidence for HTA bodies/payers decision making. Int J Technol Assess Health Care. 2025;41(1):e52. 10.1017/S0266462325100238 40524330 PMC12350084

